# Silk Fibroin/Chitosan Blended Microparticles: Preparation, Characterization, and Oil Absorption

**DOI:** 10.3390/polym18121496

**Published:** 2026-06-14

**Authors:** Ansaya Thonpho, Suchai Tanisood, Wilaiwan Simchuer, Yodthong Baimark, Prasong Srihanam

**Affiliations:** 1 Biodegradable Polymers Research Unit, Department of Chemistry and Centre of Excellence for Innovation in Chemistry, Faculty of Science, Mahasarakham University, Mahasarakham 44150, Thailand; ansaya.t@msu.ac.th (A.T.); suchai.t@msu.ac.th (S.T.); yodthong.b@msu.ac.th (Y.B.); 2 Faculty of Science and Technology, Loei Rajabhat University, Mueang District, Loei 42000, Thailand; wilaiwan.sim@lru.ac.th

**Keywords:** chitosan, emulsion, particles, oil absorption, silk fibroin

## Abstract

In this work, we extracted silk fibroin (SF) via a tertiary solvent system (CaCl_2_:Ethanol:H_2_O) and then blended it with chitosan (CS) solution to construct microparticles using the water-in-oil-emulsion–diffusion method. For the mixture of SF/CS solution aqueous phase (W) was prepared at ratios of 4:0, 3:1, 1:1, 1:3, and 0:4, using ethyl acetate as the oil phase (O). After the microparticles were prepared, their morphology was examined using scanning electron microscopy (SEM). The optimal preparation conditions were determined to be a 1% (*w*/*v*) aqueous phase with a volume of 1 milliliter, 100 milliliters of oil phase, and a stirring speed of 700 rpm. The average microparticle size was 50–100 micrometers. ATR−FTIR spectra showed unique functional groups of SF and CS, as well as interactions between the two polymers. The results of the thermal property study using a TGA instrument showed that SF microparticles had a higher maximum decomposition temperature (T*_d_*_,_*_max_*) than chitosan, and the blended microparticles’ T*_d_*_,_*_max_* increased with the proportion of SF. Most microparticles exhibited a semi-crystalline polymer structure, with SF microparticles being the most hydrophobic, followed by blended microparticles and CS, respectively. Testing for absorption capacity, the SF microparticles were more effective at absorbing used engine oil than vegetable oil and chloroform, while CS microparticles showed the highest capacity for vegetable oil. The experimental results indicated that all SF/CS blended particles played an efficiency of absorption variable by ratios of SF or CS blended. This suggested that the prepared microparticles might be useful for oil/water separation application.

## 1. Introduction

A critical worldwide issue is water pollution. There are many causes for this problem including oil and fuel spillage, industrial discharge of organic solvents, and heavy metal ions which resulted severe environmental and ecological damage [1,2,3]. It is hard to control the pollution for the long-term, and effects on aquatic ecosystems [4,5]. With previous reports, oil spill remediation such as dispersants [6], solidifiers [7], absorbents [8,9,10,11], controlled burning [12], mechanical collection [13], and biodegradation [14,15] have been widely used for oil/organic solvent cleanup. Among these strategies, using absorbents is the most promising practical use [4,16]. In general, oil absorbents properties should be composed of several advantages, including high oil sorption capacity, selective oil/water separation, fast oil sorption, low density, cheap, environmentally friendly and reusability [17,18]. Hydrophobic surfaces with interconnected microporous structure absorbent materials have been developed and proposed. Different forms of oil absorbent materials have also been reported including sponges [19,20,21], particles [22,23] and aerogels [24,25,26]. However, most of oil absorbents are inorganic or non-biodegradable materials such as graphene−based sponges [3], melamine sponges [27], and polyurethane sponges [28], which can be led to the pollution [29,30]. Therefore, it is critical to use renewable resources to produce biodegradable and environmentally friendly separation materials for oil-water separation.

Recently, natural polymers have garnered significant interest due to their biocompatibility and abundance of hydrophilic groups, which can be leveraged to engineer superhydrophilic surfaces [21,31]. This characteristic helps mitigate oil fouling during the separation process, making them highly effective candidate materials for oil/water separation [3]. Silk and chitosan are prominent examples of natural polymers that have been utilized in a variety of material designs. Raw silk fibers are composed of two primary proteins: structural “silk fibroin” (SF) and a glue-like coating protein known as “silk sericin” (SS) [32,33]. SF is predominantly composed of hydrophobic amino acid residues with fewer hydrophilic domains. Due to this unique amphiphilicity, SF exhibits considerable promise across a variety of applications [34,35,36]. For instance, studies have reported Pickering emulsions stabilized by SF nanobrushes, which are created through the self-assembly of dissolved SF triggered by silk nanofibers [37,38]. Numerous studies confirm that SF serves as an effective stabilizer for oil-in-water emulsions [39], and consequently, SF-based sorbents have attracted substantial attention for oil/water separation [40]. The micro-textures and porous features on the surfaces of silk fibers act as a natural sieve for water or oil droplets. Furthermore, the functional groups within the silk fibroin structures, such as amino, carboxyl, and hydroxyl groups, serve as ideal reaction sites for chemical modifications [41,42]. Combined with its inherent environmental friendliness and sustainability, silk is an exceptional candidate for treating oil-contaminated wastewater and oil spills. Additionally, combining silk fibers with other functional materials in various composite forms has demonstrated increasingly superior efficiency in oil/water separation [40,43,44].

Chitosan (CS), a naturally occurring cationic polysaccharide, consists of repeating D-glucosamine units joined by β-(1,4)-glycosidic bonds. It is industrially produced via the deacetylation of chitin, a primary component extracted from the exoskeleton of crustaceans [45]. CS is an abundant, inexpensive, and sustainable biopolymer with intriguing physicochemical properties [46,47]. The chemical structure of CS features abundant amino and hydroxyl groups, which impart unique functional characteristics. The primary amino groups are protonated and positively charged under appropriate conditions, allowing them to bind with negatively charged oil droplets via electrostatic attractions and/or van der Waals forces [45]. Extensive research has focused on chitosan self-assembled colloidal particles and their composites with other natural polymers [48,49]. These materials have demonstrated great potential across various fields [50], particularly when processed into CS-based three-dimensional porous sorbents [51]. However, a prominent weakness of pristine CS-based materials in oil-absorption applications is their inherently low mechanical strength and poor wet stability. Therefore, improving the structural and chemical stability of CS-based networks is essential to realize their practical, real-world application [23,52].

The development of biodegradable oil–water separation materials with high structural uniformity and balanced hydrophobic–hydrophilic properties is still a significant challenge. Although SF or CS materials have been investigated, their drawbacks, such as the high preparation complexity of SF microstructures and the low mechanical strength of CS, frequently prevent their practical use. Moreover, little research has been done on co-assembling SF and CS into structured microparticles using a simple, temperature-free water-in-oil (W/O) emulsification–diffusion technique. This work closed the gap in the structural stability of the positively charged CS matrix by effectively combining SF. The advantage of SF’s crystalline character is supporting CS strength. By using a scalable, semi-crystalline, highly customizable and environmentally benign platform for targeted oil and organic solvent absorption, it is made possible.

## 2. Materials and Methods

### 2.1. Materials

The Thai silk *B. mori* cocoons (Nang Lai variety) were derived from the Silk Innovation Center (SIC), Mahasarakham University, Khamriang sub-district, Kantharawichai, Maha Sarakham, Thailand. Sodium carbonate (Na_2_CO_3_) and calcium chloride (CaCl_2_) were supplied by Elago Enterprises Pty Ltd. (New South Wales, Australia), and ethanol (C_2_H_5_OH) and chitosan from shrimp shell Chitosan (degree of deacetylation > 85%, Mw 30–50 kDa) were purchased from Merck KGaA company (Darmstadt, Germany). Acetic acid (CH_3_COOH) was purchased from RCI Labscan Limited (Bangkok, Thailand). None of the reagent-grade chemicals used in this study needed to be further purified before being used.

### 2.2. Preparation of Chitosan Solution

The CS powder was dissolved in a 1% (*v*/*v*) acetic acid solution for 24 h to create a 2% (*w*/*v*) chitosan (CS) solution. After that, 1 M NaOH was used to bring the pH of the CS solution down to 6.5 to create CS microparticles.

### 2.3. Preparation of Silk Fibroin Solution

After being gathered and cleaned, the *B. mori* cocoons were sliced into tiny pieces. The silk sericin glue-like protein was then removed by boiling twice at 100 °C for 30 min each in a 0.5% (*w*/*v*) Na_2_CO_3_ solution. The degummed silk fibers were rinsed with distilled water until the pH was neutral before being submerged in a tertiary solvent system made up of CaCl_2_:Ethanol:H_2_O (1:2:8 by mol) for 60 min at 75 °C while being continuously stirred to produce the SF solution. The hydrolysate SF was dialyzed against distilled water for three days 133 using a dialysis membrane (Thermo Scientific, Waltham, MA, USA) to eliminate any salt. The concentration of the SF solution was then calculated and adjusted to 1% (*v*/*v*) prior to use.

### 2.4. Preparation of SF, CS and SF/CS Blended Microparticles

**Scheme 1:**
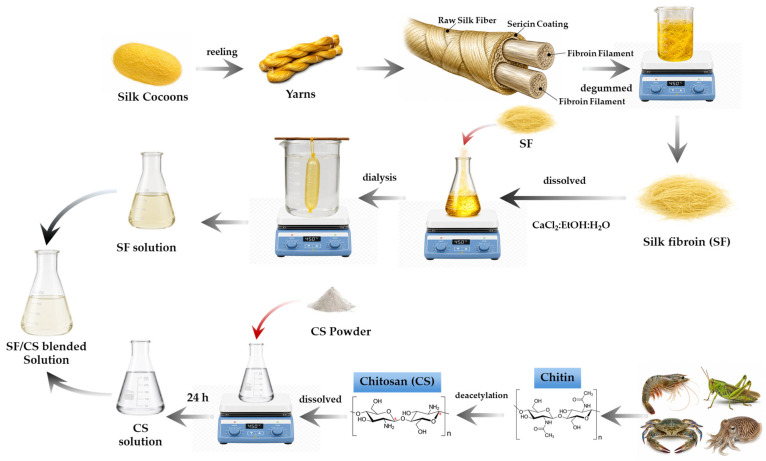
Shows diagram for SF, CS and SF/CS preparation.

**Scheme 2:**
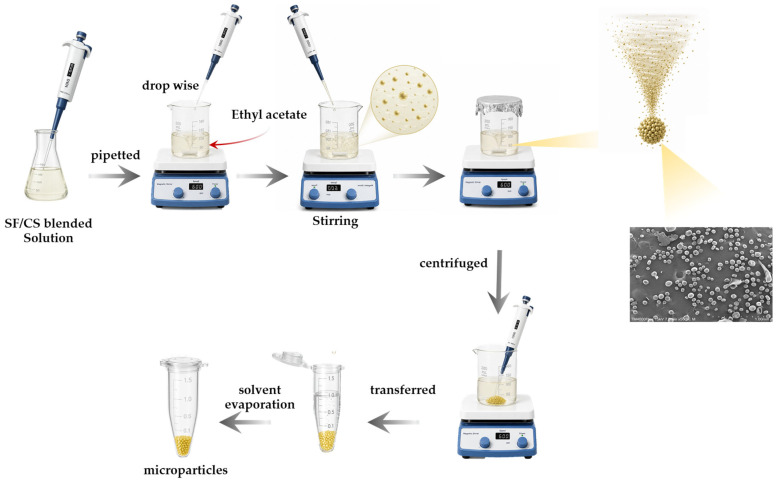
Diagram for microparticle preparation.

The water-in-oil (W/O) emulsion–diffusion process was used to manufacture all the microparticles in this work. The polymer solutions (SF, CS, and SF/CS) were represented as a water (W) phase, whereas ethyl acetate was represented as an oil (O) phase. Scheme 1 explains how to prepare SF, CS, and SF/CS solutions. It is already known that volume, concentration, stirring rate, and W:O ratios affected various forms, sizes, and properties [53]. The condition used for the particle construction was a stirring rate in the range of 600–800 rpm, concentrations at 0.25–1.00% (*w*/*v*), and 100 mL of oil phase. In the preparation step, ethyl acetate as the oil phase was firstly contained in a container that was stirred on the magnetic stirrer apparatus. In addition, Span80 was used as a surfactant. A suitable volume of each polymer solution was then gently added dropwise into the oil phase with continued stirring for 30 min. The formed particles were collected by dropper and then placed into an Eppendorf. Afterwards, the particles were separated by centrifugation. The tubes were kept in a vacuum oven at room temperature until all the ethyl acetate had evaporated. Additionally, the SF/CS blend microparticles were made using the same technique as previously mentioned. The suitable conditions of each solution were used to determine the different SF/CS blend ratios of 3/1, 1/1, and 1/3 (*v*/*v*). To achieve homogenous solutions, the blending solution was first combined and agitated for 30 min before beginning. To keep the solvent from evaporating throughout the emulsification and diffusion operations, aluminum foil was placed over the container. Scheme 2 illustrates the method of creating microparticles.

### 2.5. Characterization of the Microparticles

#### 2.5.1. Morphology Observation

The procedure outlined in Section 2.4 was used to prepare the microparticles. The samples underwent ion sputtering for gold plating after being adhered to a metal carrier stage using conductive adhesive. A scanning electron microscope (SEM) (Hitachi, TM4000Plus, Tokyo, Japan) with an accelerating voltage of 15 kV was used to examine the materials’ microstructure.

#### 2.5.2. Analysis of Interactions

A Fourier transform infrared (FTIR) spectrometer (Bruker Invenio-S, Karlsruhe, Germany) with an attenuated total reflectance (ATR) accessory was used to examine the interactions between SF and CS in the microparticles. In total, 32 cumulative scans were used to obtain the ATR-FTIR spectrum data, which had a resolution of 4 cm^−1^ and a spectral range of 4000 − 400 cm^−1^. Air was used as the reference to control this procedure. For FTIR measurement, the microparticle samples were combined with potassium bromide, crushed into a fine powder, and then compressed into pellets.

#### 2.5.3. Thermal Stability

A thermogravimetric analyzer (TGA) (SDTQ600, TA−Instrument Co. Ltd., New Castle, DE, USA) was used to examine the thermal stability of the produced microparticles. The microparticles were placed into an aluminum pan before heating in the range of 50 to 700 °C by establishing a pace of 20 °C per minute. The technique was carried out in a nitrogen atmosphere. The weight loss was noted at multiple intervals.

#### 2.5.4. Solubility Test

The microparticles’ solubility was evaluated in accordance with an earlier report [54]. Until the measured weight (W_o_) remained consistent, they were dried at 100 °C. After being put in a test tube with five milliliters of distilled water, the test samples were allowed to sit at room temperature for a whole day. The non-solubilized particles were dried in an oven for 24 h and weighed (W_f_) after 1, 3, 5, and 7 days. For every duration, the measurements were made in triplicate. The dissolution (%) values were calculated using the following Equation (1).

Dissolution (%) = [(W_o_ − W_f_)/W_o_] × 100 (1)

#### 2.5.5. X-Ray Diffraction Analysis

The produced microparticles were characterized by X-ray diffraction (XRD, Advance, Bruker D8, Karlsruhe, Germany). With Cu Kα, λ = 1.5406 Å, 40 kV, and 40 mA, the diffraction angle varied from 2θ = 5° to 60° with a step size of 0.02°/s. To quantitatively evaluate the phase variations, the relative crystallinity index (CrI, %) of each sample was monitored using the empirical Segal peak height Equation (2)

CrI (%) = I_max_ − I_am_/I_max_ × 100 (2)

where I_max_ denotes the absolute peak intensity at the maximum crystalline lattice plane and I_am_ represents the baseline intensity of the amorphous background valley, respectively.

#### 2.5.6. Oil Absorption Capacity

Following prior publication, microparticles were examined for chloroform, plant, and used engine oil [55]. Polyurethan (PU) foam was used as positive control. The process followed the previous report [56] with some modifications. After being submerged in oil that was 2 mm deep for one hour, they were centrifuged and transferred to a sieve to remove any remaining oil. Equation (3) was used to obtain the oil absorption capacity (OAC):

OAC (g/g) = W_2_ − W_1_/W_0_ (3)

where W_0_ is the initial weight of the dry microparticles (g); W_2_ and W_1_ represent the weight of the tube and the oil-absorbed pellet after centrifugation and decanting (in grams) and the weight of the tube and the dry sample (in grams), respectively.

#### 2.5.7. Microparticle Oil Absorption Cycle Test

The oil absorption cycle test was performed following previous work [56]. First, the microparticles were added to oil/water mixes in a single cycle. When the absorption approached saturation, the microparticles were removed. Manual extrusion was used to remove the oil that had been absorbed in the microparticles. Once the microparticles were unable to absorb oil, the cycle test was terminated. Equation (3) was used to calculate the oil absorption of the porous materials during the cycle by recording the mass of the microparticles following each absorption saturation and extrusion.

## 3. Results and Discussion

### 3.1. Morphological Studies

#### 3.1.1. CS Microparticles

**Figure 1:**
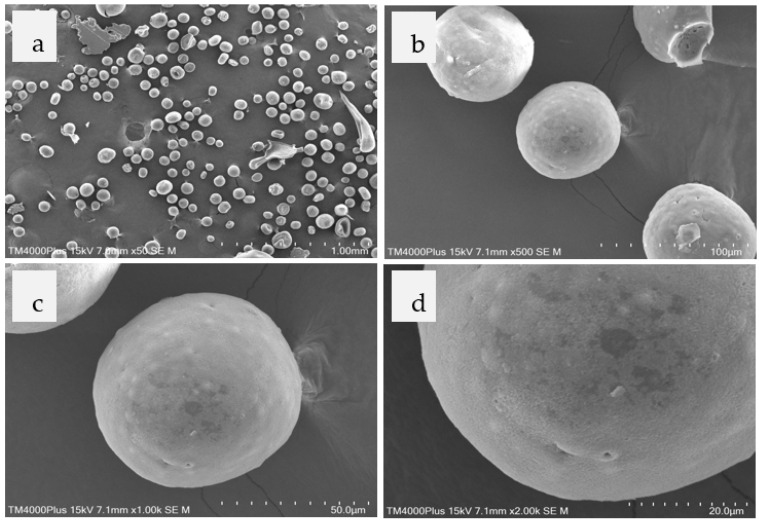
SEM micrographs of CS microparticles under different magnifications; 50× (**a**), 500× (**b**), 1000× (**c**), and 2000× (**d**), respectively.

The formation of spherical shapes in materials prepared using the water-in-oil emulsification–diffusion method is influenced by a few factors, including the volume of polymer, stirring rate, the water (W) to oil (O) phase ratio, and surfactants or crosslinking agents [53,57,58]. The microparticles’ spherical shape offers several benefits, particularly in terms of the surface that encounters the material to be absorbed and the direction of release [58,59]. The shape of the produced 1% CS microparticles is shown in Figure 1. The size of the prepared microparticles is between 60 to 100 μm. Under low magnification (Figure 1a,b), the microparticles have a relatively uniform size and are spherical. The microparticles are closely packed and have a smooth surface with no evident fissures or roughness when the magnification is raised (Figure 1c). This may indicate that CS’s internal structure created a chemical connection [53]. When considering the maximum magnification (Figure 1d), voids appear distributed within the microparticles, and there are small holes on the surfaces of the particles. This characteristic is believed to be caused by the water evaporation [60], which was the solvent used in preparing the CS solution.

#### 3.1.2. SF Microparticles

**Figure 2:**
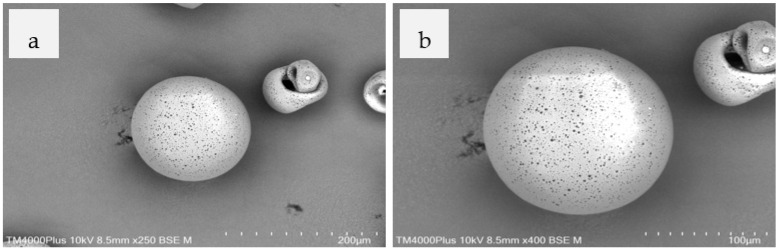
SEM micrographs of SF microparticles with different magnifications; 250× (**a**) and 400× (**b**), respectively.

An SEM micrograph of SF microparticles is displayed in Figure 2. The chosen SF microparticles typically have a solid texture, smooth surfaces, and a spherical form (Figure 2a). Nevertheless, it is currently challenging to prepare the SF microparticles to have a spherical shape. Most of the shapes that are produced are unfinished, giving rise to a variety of morphologies such as porous particles, lumps, and thin sheets. These characteristics are expected to result from using old silkworm cocoons to prepare a silk fibroin solution. The structure or arrangement of amino acids in the silk protein may be damaged or altered, affecting the formation of chemical bonding or spherical shapes when compared to previous experimental results [53,57,59]. SF microparticles, which range in size from 120 to 150 μm, are around 1.5 times larger than CS microparticles. Higher magnification reveals many tiny pores that are uniformly dispersed throughout the particle’s surface, as seen in Figure 2b. These pores are thought to be created as water evaporates during the particle production procedure. The evaporation of water causes the SF molecules to come closer together, resulting in the formation of chemical bonds. These bonds resulted in the particle being harder and more solid [61,62].

#### 3.1.3. SF/CS Blended Microparticles

The SF/CS blended microparticles were prepared using 1% (*w*/*v*) CS solution and 1% SF solution with different volume ratios of 3:1, 1:1, and 1:3, respectively. The selection of these ratios is grounded in the need to systematically study how varying the proportion of SF and CS affects the physicochemical and biological properties of the resulting mixed materials. These ratios represent a spectrum of SF-dominant (3:1), balanced (1:1), and CS-dominant (1:3) compositions. Our study revealed that the blending ratios significantly influenced properties such as mechanical strength, degradation rate, porosity, and biological properties [53]. There is no universally optimal ratio; instead, the best ratio depends on the intended application.

**Figure 3:**
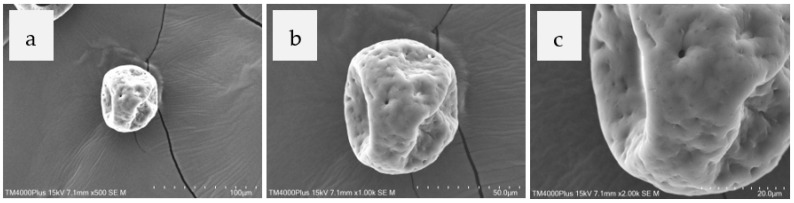
SEM micrographs of SF/CS (3:1) blended microparticles with different magnifications; 500× (**a**), 1000× (**b**), and 2000× (**c**), respectively.

Figure 3 shows the SF/CS blended microparticles at a 3:1 ratio. At low magnifications (Figure 3a,b), the shapes of the microparticles obtained from this ratio are not spherical, with irregular form and indentations, and have a comparatively smooth surface. Sizes range from 30 to 60 μm on average. However, the particle preparation is found to be modest and incomplete at this ratio. This might be caused by the greater SF ratio, which prevents the creation of entire particles. SF would be packed together before interaction with CS. Compared to the pure SF microparticles (Figure 2), the CS helps with the particles’ wither thorough formation. The surface of the microparticles is rough due to the unevenness of the surface area when considered under high magnification (Figure 3c).

**Figure 4:**
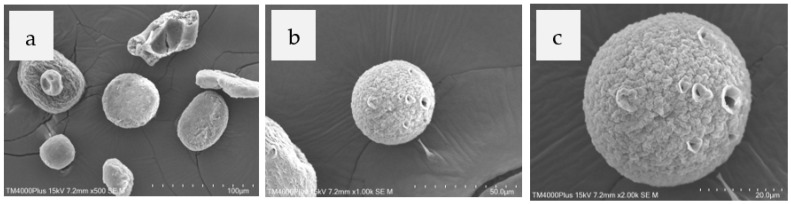
SEM micrographs of SF/CS (1:1) blended microparticles with different magnifications; 500× (**a**), 1000× (**b**), and 2000× (**c**), respectively.

Figure 4 shows the SF/CS blended microparticles at a 1:1 ratio. The average particle size ranges from 20 to 60 μm on average. Under low magnification (Figure 4a), the prepared microparticles have different shapes: rod, round, flat, ellipse, and incomplete round shape. At high magnifications (Figure 4b,c), the microparticles are relatively complete, with some pores on the surface. This pore is caused by polar components of the microparticle which evaporated during drying [63]. The obtained results also indicate that addition of CS helps to form more complete particles compared to pure SF.

**Figure 5:**
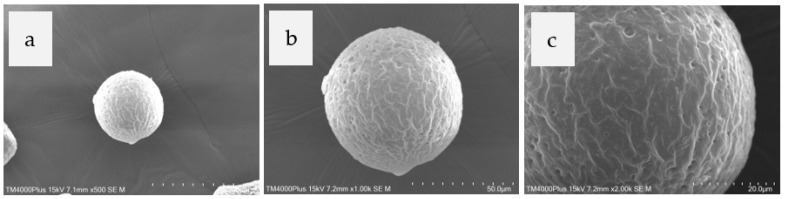
SEM micrographs of SF/CS (1:3) blend microparticles with different magnifications; 500× (**a**), 1000× (**b**), and 2000× (**c**), respectively.

Figure 5 shows the SF/CS blended microparticles at a 1:3 ratio. The prepared particles are spherical, are well-formed, and have a relatively smooth surface. The average size of the microparticles is between 60 and 80 μm (Figure 5a,b). Generally, many spherical microparticles can be prepared from this ratio. However, it is found that the surface of the microparticles have a wavy texture when observed at high magnification (Figure 5c). This is believed to be due to the difference polarity between the SF and CS, resulting in an uneven compatibility.

**Figure 6:**
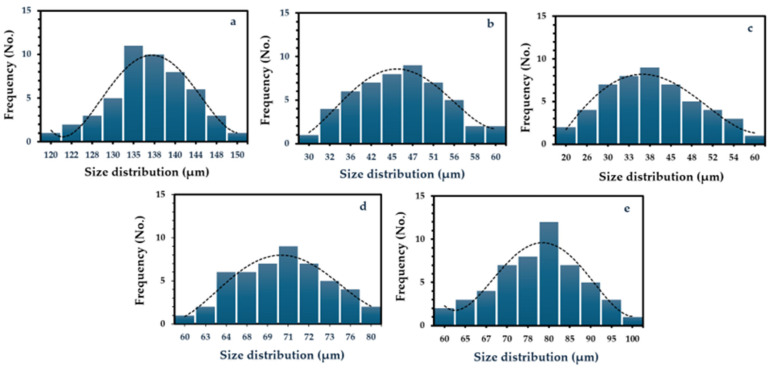
Microparticle size distribution counted from SEM images; SF (**a**), SF/CS blended at 3:1 (**b**), 1:1 (**c**), and 1:3 (*v*/*v*) (**d**) ratios, and CS (**e**).

Previous reports suggested that the sizes and shapes of the blended microparticles are affected by several factors, particularly the polymer chain, which is derived from the preparation process. Additionally, if different types of polymers are used, the blending ratio is another factor [53,64]. The particle size distribution could be summarized in Figure 6.

### 3.2. Water Solubility

The water solubility values (% weight loss) for the prepared microparticles are shown in Table 1. The results show that all prepared microparticles could exist in water for over seven days, which indicated the stability of the microparticles. At the final time, all microparticles remained at over 75 percent of their weight. Among types, SF/CS blended at 3:1 and 1:3 showed gradual degradation starting on day 3 and then gradually lost weight until the end of the experimental test. Statistical analysis indicated that there was no significant difference (*p >* 0.05) in water dissolution percentages among native SF, native CS, and their various blends across all tested intervals, validating that all particle types possessed equivalent high-water stability exceeding 75% up to 7 days. CS microparticles showed stability in water like SF. This is due to CS being generally non-degradable in water. SF/CS blended at 1:1 and was also found to be stable in water. This suggests that this ratio supported chemical bond formation between both materials, enhancing water solubility [65].

### 3.3. Functional Group Analysis

**Figure 7:**
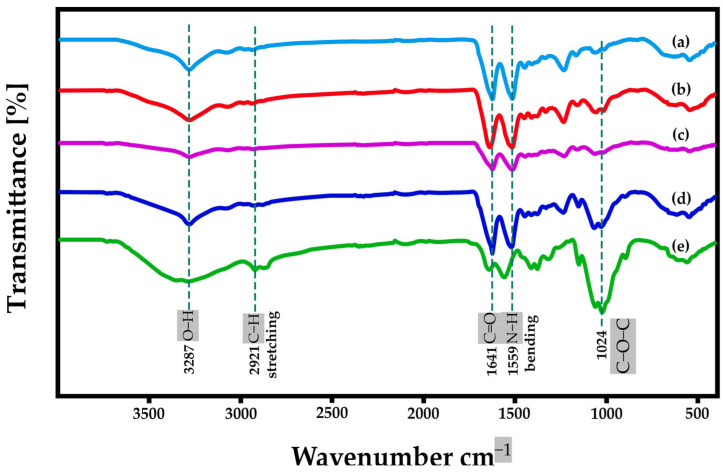
ATR−FTIR spectra of microparticles; native SF (**a**), SF/CS blended at 3:1 (**b**), 1:1 (**c**), and 1:3 (*v*/*v*) (**d**) ratios, and native CS (**e**).

Functional group analysis is a common use of ATR-FTIR spectroscopy. The absorption peaks of SF microparticles are displayed in Figure 7a. Peptide bonds are the areas on proteins where amino acids are joined to one another. Amide I (1700 − 1600 cm^−1^), Amide II (1600 − 1500 cm^−1^), and Amide III (1300 − 1200 cm^−1^) make up this region, which is referred to as “amide types” [53,66,67]. The three different kinds of amides are associated with the protein’s secondary structure, such as the α-helix or β-pleated sheet. According to the spectra, amide I (C=O stretching) has absorption areas at 1640 cm^−1^ (α−helix), 1620–1640 cm^−1^ (β−turns), and 1645 cm^−1^ (random coil). Moreover, amide III has an absorption area (C−N and N−H bending) at 1325 cm^−1^ and amide II (N−H bending and C−N stretching) has an absorption area at 1558 cm^−1^ [66,68].

The CS microparticles (Figure 7e) show absorption in the range of 3291–3361 cm^−1^, which corresponds to the locations of hydrogen bond formation inside the molecule and the N−H and O−H stretching groups. The absorption sites at roughly 2921 and 2877 cm^−1^ are caused by asymmetric stretching and C-H stretching, respectively. These absorption locations are unique to polysaccharides, which are also frequently present in other polysaccharides such as carrageenan [69], xylan [70], and glucan [71]. The asymmetric stretching of the glycosidic bond (C−O−C linkage) is represented by the absorption band at 1153 cm^−1^, while the C−O stretching groups are represented by the absorption regions at 1066 and 1028 cm^−1^. The previously described structure of chitosan is indicated by all the absorption areas [72,73].

The SF/CS microparticles blended at a ratio of 3:1 (Figure 7b) exhibits an overall absorption characteristic like both pure SF and CS microparticles. However, slight differences in absorption are observed, particularly in the absorption regions at 3700 cm^−1^ and 1158–1167 cm^−1^. These differences are expected to arise from interactions between the functional groups of the SF (C=O, N−H) and CS (N−H, O−H, C−O−C) via hydrogen bonds and electrostatic interactions.

Figure 7c shows the absorption peaks of SF/CS blended microparticles at a 1:1 ratio. The peaks associated with SF begin to decrease, while the peaks of CS become more dominant (3700–3720 cm^−1^ and 1158–1167 cm^−1^). However, at the absorption position around 1580 cm^−1^, there is a clear shift toward lower absorption values. This indicates that the amine groups (−NH_2_) of CS are transforming into amide II (−NH) of SF, as well as the formation of bonds between the hydroxyl groups (−OH) of CS and the peptide positions of SF.

The absorption peaks of SF/CS blended microparticles at a 1:3 ratio are displayed in Figure 7d. The absorption pattern is like that of CS, especially in the regions around 1620–1630 cm^−1^ (C=O group), 100–1100 cm^−1^ (asymmetric position by β−(1–4) glycosidic linkages) [74], and 1560 cm^−1^ (−NH group). The creation of a chemical link between SF and CS is what causes the change in the absorption zones. Table 2 provides a summary of the functional groups’ absorption peaks.

### 3.4. Weight Loss Analysis

**Figure 8:**
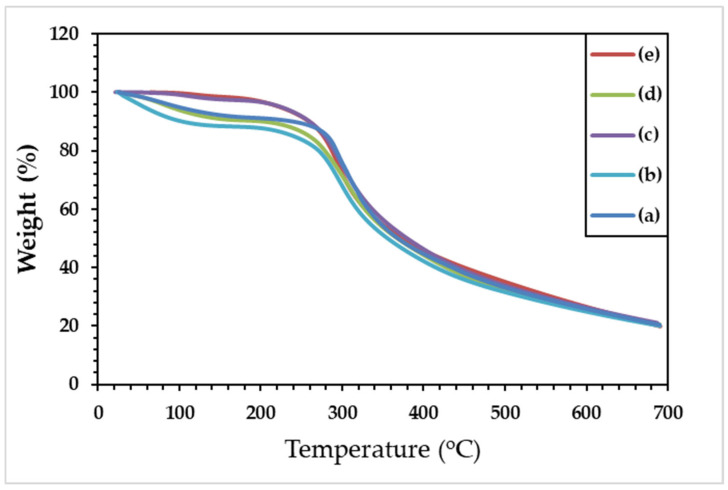
TG curves of microparticles; native SF (**a**), SF/CS blended at 3:1 (**b**), 1:1 (**c**), and 1:3 (*v*/*v*) (**d**) ratios, and native CS (**e**).

**Figure 9:**
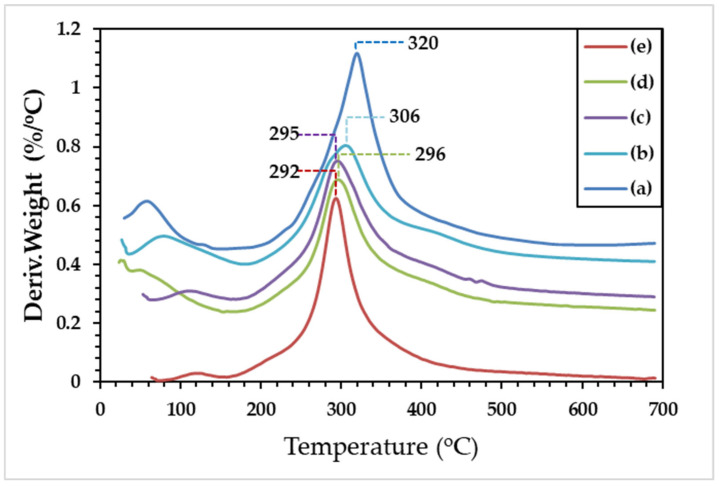
**D**TG curves of microparticles; native SF (**a**), SF/CS blended at 3:1 (**b**), 1:1 (**c**), and 1:3 (*v*/*v*) (**d**) ratios, and native CS (**e**).

The TG curves for each prepared microparticle are displayed in Figure 8. At least two stages of degradation occur in the microparticles: the first occurs at about 100 °C, where the evaporation of water molecules causes the microparticles’ weight to significantly decrease [56]. The degradation of the primary polymer structure takes place in the second stage at temperatures between 300 and 400 °C [60,61,75]. Compared to other particles, CS microparticles exhibit greater resistance to deterioration. The weight of microparticles (charred residues) stays at roughly 25% at the highest temperature test. The remaining substances are carbon that cannot be degraded [53]. At the final temperature test, the content of the blended microparticles’ charred residue is comparable to that of native SF. The maximum amount of charred residue was found in the native CS microparticle. Additionally, compared to other microparticles, the native CS microparticle was more heat stable. The maximum temperature decomposition (T*_d_*_,_*_max_*) could be detailed in the DTG curves as shown in Figure 9. Table 3 provides the microparticle’s decomposition. The values of T5%, T10%, and T50% of native CS microparticles are higher than native SF microparticles. This indicates that the SF microparticles absorbed water at higher rates than CS microparticles. In the case of blended SF/CS, the 1:1 ratio shows the highest value. It is suggested that this ratio might be formed from chemical bonding between SF and CS, resulting in decreased water absorption [53,76,77]. However, the values of T5% and T10% for SF/CS blended at 1:3 were higher than for SF/CS blended at 3:1. The results reveal that at low temperatures, the SF/CS blended at 3:1 decomposed rapidly, but it decomposed slowly at higher temperatures. The T*_d_*_,_*_max_* of the SF/CS blended microparticles, increased with the SF content.

### 3.5. Crystallinity Determination

**Figure 10:**
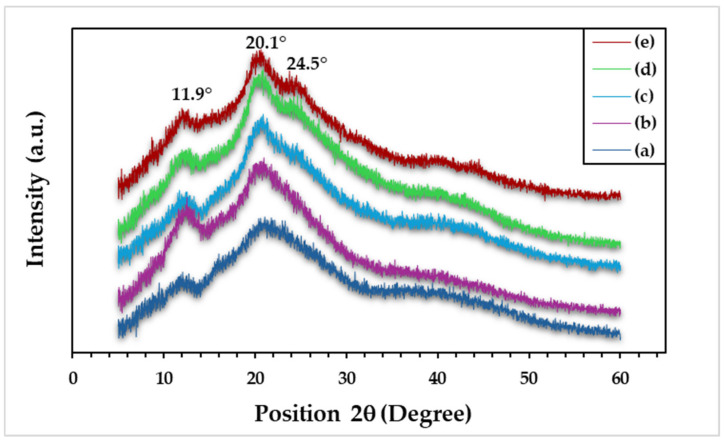
XRD patterns of microparticles; native SF (**a**) SF/CS blended at 3:1 (**b**), 1:1 (**c**), and 1:3 (*v*/*v*) (**d**) ratios, and native CS (**e**).

Crystaline structure of the microparticles was analyzed using X-ray diffraction (XRD) by scanning in the 2θ range of 5–60°. As shown in Figure 10, it was found that all the prepared microparticles exhibited XRD signals characteristic of a semi-crystalline polymer, within the main peaks appearing at approximately 11.9° and 20.6° (Figure 10). These peaks are specific to the β−sheet structure of SF [53]. In addition, broad peaks were observed at approximately 20°, which are associated with the crystalline regions of CS [78,79]. For the SF/CS microparticles blended at different ratios (Figure 10b–d), sharper and more intense peaks gradually inclined to CS depending on CS ratios. This suggests that the crystallinity of the blended microparticles is dependent on both SF and CS contents. Generally, chitosan is a semicrystalline polymer. The preparation process and form as well as the intermolecular H−bonding are main factors in its crystalline structure. When blending SF, the SF/CS microparticles revealed sharp and more intense peak signals, indicating an increase in the degree of crystallinity. The hydrophobic parts in the SF structure might be from the β−sheet structure and promote the formation of hydrogen bonds between the amino groups of the SF and the hydroxyl groups of CS [80]. Table 4 shows the relative crystallinity index (CrI) of the prepared microparticles. As illustrated in Table 4, pure CS displays a characteristic broad diffraction peak centered at 2θ = 21.38°, indicative of its semi−crystalline nature with a moderate CrI of 37.36%. On the other hand, the pure SF diffractogram exhibits a prominent peak at 2θ = 20.62°, signifying the dominant presence of a highly organized stable crystalline structure. This is commonly categorized as Silk II conformation with CrI of 41.92%. Additionally, SF revealed a sharp characteristic reflection at a lower angle of 2θ = 10.21° (CrI = 42.35%), which corresponds to the less stable Silk I polymorph.

Considering blended microparticles, a distinct and systematically coordinated peak shift was observed across the multi-component composites. The primary diffraction peak shifted systematically between the coordinates of the pure components, converging towards 2θ = 20.87° for the 3:1 blend and 20.86° for the 1:1 blend and migrating further rightward to 20.95° as the CS content became heavily dominant in the 1:3 formulation. This smooth, non-linear migration into a unified singular peak mathematically substantiates the excellent miscibility and structural homogeneity of the SF/CS matrix. It implies a strong molecular co-assembly rather than a macro-phase separation. This is driven primarily by extensive intermolecular hydrogen bonding networks forming between the protonated amine/hydroxyl groups of CS and the carbonyl/amide segments along the SF backbone. Interestingly, the CrI of the series followed a non-linear parabolic trend, reaching its peak of 44.78% at the equitable 1:1 ratio and maintaining an elevated value of 43.29% at the 3:1 ratio. This remarkable increase in CrI relative to both raw components provides definitive evidence of a synergistic crystallization effect. The structural incorporation of minor fractions of CS chains within a major SF network (3:1), or vice versa, effectively serves as a structural nucleating template. This interlocking molecular interface restricts the chaotic thermal motion of random amorphous coils, thereby promoting a more ordered spatial orientation into co-crystalline matrices. Conversely, SF/CS 1:3 caused a drop in crystallinity down to 35.89%. This decrease is attributed to the steric hindrance imposed by the bulky polysaccharide structures of CS, which overpowers the system and suppresses the native β-sheet nucleation of the minor SF portion.

### 3.6. Absorption Capacity Test

The oil and organic solvent absorption capacities (OAC) of native SF, native CS, and their blended variants (SF/CS) were evaluated gravimetrically using chloroform, plant oil, and used engine oil. A commercial polyurethane (PU) foam served as a high−performing positive reference control to validate the experimental methodology. The gravimetric absorption capacities (g/g) for all samples are compiled in Table 5. The positive control, PU foam, exhibited the highest performance across all tested fluids, yielding absorption capacities of 35.5 ± 1.0 g/g for chloroform, 30.4 ± 1.0 g/g for plant oil, and 36.6 ± 2.0 g/g for used engine oil. Among the biopolymer matrices, a non-linear performance trend was observed based on the blending ratio of silk fibroin to chitosan. Native SF and native CS microparticles demonstrated modest capacities, particularly for chloroform (4.6 ± 0.2 g/g and 4.3 ± 0.2 g/g, respectively). Notably, incorporation of both polymers revealed a significant synergistic effect. The SF/CS (1:1) blend achieved peak performance across all fluids, absorbing 9.6 ± 0.3 g/g of chloroform, 24.8 ± 0.3 g/g of plant oil, and 26.6 ± 0.2 g/g of used engine oil. This represents roughly a two-fold increase in chloroform absorption and an approximate 35% to 85% increase in plant oil absorption compared to the respective homopolymers. Deviations from this equal ratio, such as in the SF/CS (3:1) and SF/CS (1:3) formulations, resulted in a systematic decline in uptake capacity across all media. Furthermore, the fluid type strongly dictated gravimetric retention. For almost all biopolymer compositions, the absorption capacity followed the trend of Used Engine Oil > Plant Oil > Chloroform. This trend is inversely proportional to fluid density, where the densest solvent (chloroform) yielded the lowest gravimetric retention. The CS structure has many amino groups (–NH_2_) that dissociate to form positive charge in the acid condition, which can create adhesive forces with the negatively charged molecules of vegetable oil at the carboxylic group. Moreover, the surface characteristics of microparticles, such as roughness or inward concavity, may increase the contact area, leading to higher absorption [81]. The remarkable, synergistic peak observed for the SF/CS (1:1) blend suggests a highly balanced intermolecular network. At this specific stoichiometric ratio, the strong electrostatic interactions between the cationic amine groups of chitosan and the anionic carboxyl groups of silk fibroin likely produce an ideal cross-linking density. This robust interfacial bonding prevents structural collapse or shrinkage when immersed in fluids, preserving a high pore volume and interconnectivity that facilitates enhanced capillary action. When either polymer dominates the matrix (3:1 or 1:3 ratios), phase separation or unentangled polymeric domains may occur, resulting in localized pore collapse or restricted volume space. Therefore, it is expected that the SF/CS blended microparticles can be applied as oil absorbents in the environment when combined with other devices or materials.

**Figure 11:**
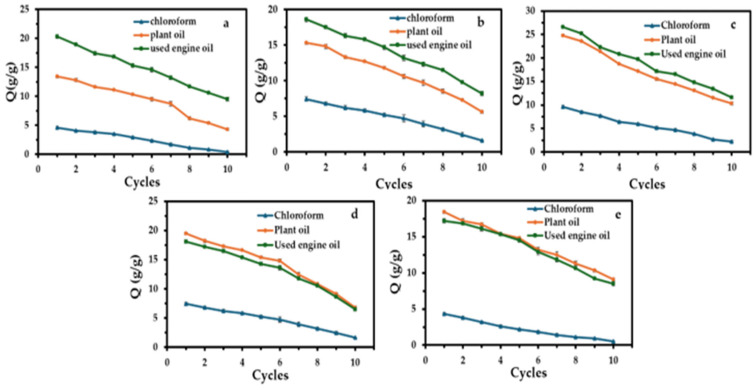
Oil absorption cycles of microparticles; native SF (**a**), SF/CS blended at 3:1 (**b**), 1:1 (**c**), and 1:3 (*v*/*v*) (**d**) ratios, and native CS (**e**).

Figure 11 shows the oil absorption capacity (Q) of all prepared microparticles across ten consecutive cycles using three distinct fluids: chloroform, plant oil, and used engine oil. This trend suggests that the highly viscous and hydrophobic nature of hydrocarbons (used engine oil and plant oil) promotes better capillary entrapment within the porous SF framework compared to the highly volatile organic solvent chloroform. Over the course of 10 cycles, all samples experienced a continuous decline in absorption capacity, indicating structural fatigue or residual fluid entrapment within the SF matrix. In the initial cycle, the CS (Figure 11e) exhibited a clear selectivity based on the fluid type. Plant oil achieved the highest maximum capacity of 18.4 ± 0.2 g/g, followed by used engine oil, which demonstrated a highly comparable initial capacity of 17.2 ± 0.3 g/g. Chloroform displayed the lowest initial uptake at 4.3 ± 0.2 g/g. The significantly higher capacities for plant oil and used engine oil can be attributed to their hydrophobic aliphatic structures and higher viscosities, which favor capillary−driven physical entrapment within the porous network of the CS matrix without damaging the framework. The dramatic performance drops when using chloroform highlights its adverse effects on the material. Chloroform, as an aggressive organic solvent, likely causes partial dissolution or structural collapse of the hydrophobic surface coatings or polymer networks within the CS microparticles during the recovery phase. The superior performance of the SF/CS (3:1) blended microparticles (Figure 11b) toward viscous hydrocarbons (used engine oil and plant oil) is attributed to the synergistic interaction between the two biopolymers. The high proportion of SF provides a mechanically stable, open three−dimensional crystalline framework, while CS provides structural crosslinking. This combination creates optimal surface tension and capillary forces within the porous matrix, enabling the efficient entrapment of high-viscosity oily molecules. Used engine oil exhibited the highest initial uptake at 18.6 ± 0.3 g/g, followed by plant oil (15.3 ± 0.2 g/g), and chloroform demonstrated the lowest absorption capacity at 7.4 ± 0.3 g/g. The SF/CS (1:1) microparticles (Figure 11c) exhibited remarkably high initial capacities across all tested liquids, maintaining a distinct fluid selectivity. The highest absorption performance was achieved at 26.6 ± 0.2 g/g for used engine oil. Plant oil demonstrated a similarly excellent capacity of 24.8 ± 0.3 g/g. The lowest initial uptake at 9.6 ± 0.3 g/g displayed for chloroform. Notably, the initial capacities for the 1:1 composite surpass those of both pure CS and the SF/CS (3:1) variant. This significant boost indicates that an equal ratio of SF to CS yields an optimized micro−porous geometry. The balanced interaction between the beta-sheet structures of SF and the highly crosslinked network of CS establishes an ideal structural density and rough−texture surface, expanding the available internal volume for physical capillary fluid entrapment. Despite the gradual decay, the SF/CS (1:1) composite retains double−digit absorption capacities for both oil types at the final cycle, confirming robust cyclical viability for oily waste remediation. During the first cycle, the SF/CS (1:3) showed high initial affinity toward lipid-based liquids. Plant oil reached the highest initial uptake at 19.5 ± 0.2 g/g, followed by used engine oil at 18.1 ± 0.3 g/g, and chloroform demonstrated the lowest at 7.4 ± 0.3 g/g (Figure 11d). The dominant presence of CS provides a rigid polymer matrix. This structural rigidity supports a steady oil uptake via surface tension interaction. The hydrophobic domains within the minor SF fraction still assist in attracting non-polar hydrocarbons into the pore structures. The higher CS resulted in a higher percentage capacity loss for both oils compared to the 1:1 and 3:1 variant. This indicates a less flexible matrix under repeated regeneration forces.

## 4. Conclusions

This study successfully synthesized biodegradable SF, CS, and SF/CS blend microparticles using a water-in-oil emulsification–diffusion technique. Intermolecular interactions, such as hydrogen bonding and electrostatic cross-linking between the silk fibroin and chitosan matrices, enhanced the overall crystallinity and thermal stability of the composite structures, yielding excellent water stability across all formulations. The variations in surface hydrophobicity and chemical functional groups between the protein and polysaccharide networks led to highly selective oil adsorption behavior. Consequently, these natural, biopolymer-based microparticles offer a highly tunable, eco-friendly platform for targeted oil spill remediation and environmental aquatic cleanup. In the future, we will focus on the following key perspectives to transition these materials toward practical application: adsorption kinetics and mechanics, structural optimization, composite functionalization, and recyclability and field testing.

## Figures and Tables

**Table 1:** Summary of the water solubility of different types of microparticles.

Types	Dissolution Time (Days)				
	0	1	3	5	7
Native SF	100 ± 0.00	100 ± 0.00	90.00 ± 3.00	85.00 ± 2.00	80.00 ± 3.00
SF/CS (3:1)	100 ± 0.00	100 ± 0.00	88.00 ± 2.00	82.00 ± 2.00	75.00 ± 2.00
SF/CS (1:1)	100 ± 0.00	100 ± 0.00	90.00 ± 3.00	85.00 ± 4.00	80.00 ± 2.00
SF/CS (1:3)	100 ± 0.00	100 ± 0.00	86.00 ± 2.00	80.00 ± 2.00	76.00 ± 1.00
Native CS	100 ± 0.00	100 ± 0.00	92.00 ± 3.00	84.00 ± 4.00	79.00 ± 3.00

**Table 2:** Summary of the absorption peaks in the FTIR spectra of different types of microparticles.

Types	Absorption (cm^−1^)				
	O−H Str.	C−H Str.	C=O Str.	N−H Str.	C−O Blend
Native SF	3290	2921	1641	1558	−
SF/CS (3:1)	3290	2928	1642	1559	1024
SF/CS (1:1)	3290	2956	1623	1559	1024
SF/CS (1:3)	3290	2956	1624	1559	1024
Native CS	3291	2921	1645	1589	1024

**Table 3:** Thermal decomposition properties of different microparticles.

Types	Thermal Decomposition (°C)				^1^ Charred Residues (%)
	^1^ T_5%_	^1^ T_10%_	^1^ T_50%_	^2^ T*_d_*_,_*_max_*	
Native SF	98	238	386	320	28
SF/CS (3:1)	88	201	387	312	29
SF/CS (1:1)	224	260	401	300	29
SF/CS (1:3)	221	253	373	297	29
Native CS	222	259	424	294	30

**^1^** Obtained from TG thermograms. **^2^** Obtained from DTG thermograms.

**Table 4:** XRD peak positions, amorphous intensities, and calculated crystallinity index (CrI) of SF, CS, and their blend matrices.

Samples	Crystalline Max 2θ (°)	Peak Intensity (I_max_)	Amorphous Valley (I_am_)	Crystallinity Index (CrI,%)
Pure CS	21.38	1507.00	944.00	37.36
SF/CS (1:3)	20.95	1474.00	945.00	35.89
SF/CS (1:1)	20.86	1541.00	851.00	44.78
SF/CS (3:1)	20.87	1684.00	955.00	43.29
Pure SF	20.62	1441.00	837.00	41.92

**Table 5:** Absorption capacity of the different microparticles.

Types	Absorption Capacity (g/g)		
	Chloroform	Plant Oil	Used Engine Oil
Native SF	4.6 ± 0.2	13.4 ± 0.2	20.3 ± 0.3
SF/CS (3:1)	7.4 ± 0.3	15.3 ± 0.3	18.6 ± 0.3
SF/CS (1:1)	9.6 ± 0.3	24.8 ± 0.3	26.6 ± 0.2
SF/CS (1:3)	7.4 ± 0.3	19.5± 0.2	18.1 ± 0.3
Native CS	4.3 ± 0.2	18.4 ± 0.2	17.2 ± 0.3
PU foam	35.5 ± 1.0	30.4 ± 1.0	36.6 ± 2.0

## Data Availability

The raw data supporting the conclusions of this article will be made available by the authors on request.
